# Neurodegenerative Diseases: From Dysproteostasis, Altered Calcium Signalosome to Selective Neuronal Vulnerability to AAV-Mediated Gene Therapy

**DOI:** 10.3390/ijms232214188

**Published:** 2022-11-16

**Authors:** Tam T. Quach, Harrison J. Stratton, Rajesh Khanna, Sabrina Mackey-Alfonso, Nicolas Deems, Jérome Honnorat, Kathrin Meyer, Anne-Marie Duchemin

**Affiliations:** 1 Institute for Behavioral Medicine Research, Wexner Medical Center, The Ohio State University, Columbus, OH 43210, USA; 2 INSERM U1217/CNRS UMR5310, Université de Lyon, Université Claude Bernard Lyon 1, 69677 Lyon, France; 3 Department of Pharmacology, University of Arizona, Tucson, AZ 85716, USA; 4 Department of Molecular Pathobiology, New York University, New York, NY 10010, USA; 5 French Reference Center on Paraneoplastic Neurological Syndromes and Autoimmune Encephalitis, Hospices Civils de Lyon, 69677 Lyon, France; 6 SynatAc Team, Institut NeuroMyoGène, 69677 Lyon, France; 7 The Research Institute of Nationwide Children Hospital, Columbus, OH 43205, USA; 8 Department of Pediatric, The Ohio State University, Columbus, OH 43210, USA; 9 Department of Psychiatry and Behavioral Health, The Ohio State University, Columbus, OH 43210, USA

**Keywords:** neurodegeneration, dysproteostasis, calcium signaling, dendritic dystrophy, gene therapy, neuronal vulnerability, CRMP3/DPYSL4

## Abstract

Despite intense research into the multifaceted etiology of neurodegenerative diseases (ND), they remain incurable. Here we provide a brief overview of several major ND and explore novel therapeutic approaches. Although the cause (s) of ND are not fully understood, the accumulation of misfolded/aggregated proteins in the brain is a common pathological feature. This aggregation may initiate disruption of Ca^++^ signaling, which is an early pathological event leading to altered dendritic structure, neuronal dysfunction, and cell death. Presently, ND gene therapies remain unidimensional, elusive, and limited to modifying one pathological feature while ignoring others. Considering the complexity of signaling cascades in ND, we discuss emerging therapeutic concepts and suggest that deciphering the molecular mechanisms involved in dendritic pathology may broaden the phenotypic spectrum of ND treatment. An innovative multiplexed gene transfer strategy that employs silencing and/or over-expressing multiple effectors could preserve vulnerable neurons before they are lost. Such therapeutic approaches may extend brain health span and ameliorate burdensome chronic disease states.

## 1. Introduction

Uncovering the specific/common mechanisms underlying the disruption pathways among ND is critical to both basic and applied neuroscience. These findings will contribute to the identification of the neural systems underlying not only our perception, emotions, and cognition under the physiological condition with which we think, act, and react but also highlight the pathological perturbations of neural connectomic networks observed in most well-known major ND displaying impairment in memory and cognition such as Alzheimer (AD), Huntington (HD), Creutzfeldt-Jakob (CJD), Parkinson (PD)’s diseases, and Amyotrophic Lateral Sclerosis (ALS). Amongst many pathological processes, the main advantage of the shared molecular event is that neuronal plasticity involved in cognition/learning/memory, affected during aging and ND, may have some conserved anomalies, especially in the medial temporal lobe network, with maladaptation of immune responses [1], dysproteostasis [2], altered regulation of calcium signaling [3] combined with impaired mitochondrial [4]/Endoplasmic Reticulum (ER) activities [5], and dendritic dystrophy. Together, they may offer novel research avenues for exploring “disease-specific” and “disease-disease overlapping” pathogenic mechanisms as ND patients often have specific, as well as common clinical symptoms which may generate from a “shared-pan-neurodegenerative proteomic” (Table 1). It is crucial to identify upstream events—specific and/or common causes—initiating dystrophy of dendrites, synaptic dysfunction, selective neuronal degeneration, and specific neural clusters across ND [6]. These processes are critical for gene therapy which has emerged as a transformative alternative to bypass the pleiotropic effects of small standard molecule treatment because it enables selectively targeting specific proteins, neuronal types, or brain regions [7].

## 2. Endoplasmic Reticulum Stress (ERS) and Dysproteostasis in Major Neurodegenerative Diseases

A common clinical hallmark of ND is dementia. This is associated with the deposition of insoluble proteinaceous inclusions in and around affected neurons throughout the brain with altered spines/dystrophic dendrites [105]. The primary insoluble constituent is often a disease-specific protein, such as amyloid-β (Aβ) and tau in AD, α-synuclein (α-syn) in PD, Zn-Cu superoxidase dismutase (SOD1)/Fused in Sarcoma RNA Binding Protein (FUS) in ALS, Huntingtin (Htt) in HD, and prions in CJD. There is also evidence that in autoptic brain samples from AD, HD, and PD patients [106], altered expression patterns of Heat Shock Protein Family A Member 2 (HSPA2), DNA Heat Shock Protein Member B2 (DNAJB2), Translocase of outer mitochondrial membrane 70A (TOMM70A) can be observed. In addition, there is an emerging role for dysfunctional interactions between proteins and their effectors thought to contribute to multiple ND states. For example, proteins fragmented by calpains, such as Transactive response DNA Binding Protein 43 (TDP-43) [107], which is involved in an expanding spectrum of ND, can contribute to neuronal dysfunction. Not surprisingly, such dysfunctional protein relationships have been observed in AD, PD, ALS, HD, and CJD.

**Figure 1:**
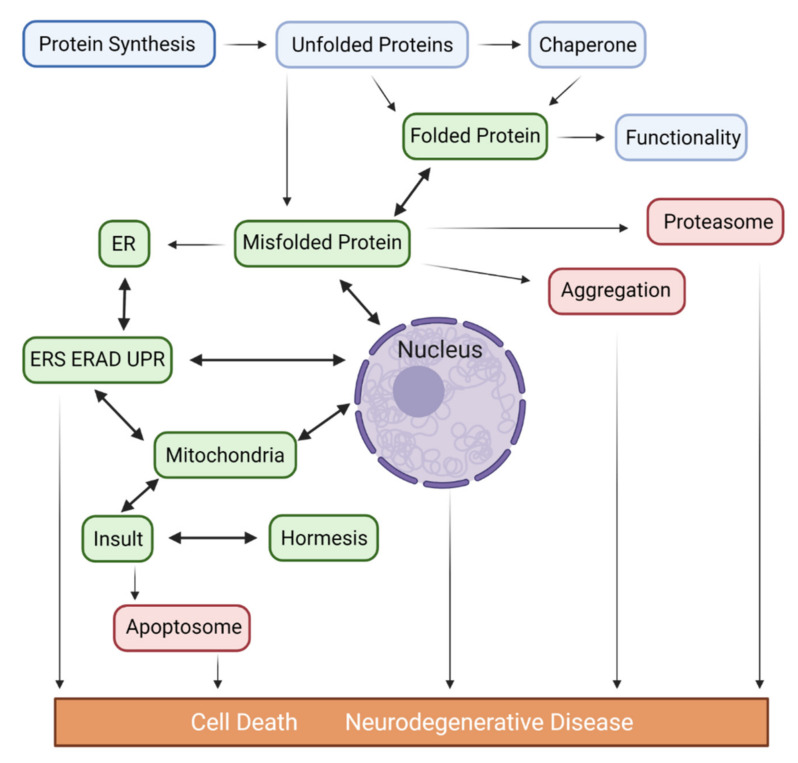
Proposed pathways depicting the potential role of misfolded proteins and the contribution of ER/Mitochondria/Nucleus in the pathogenesis of major ND. Accumulated unfolded/misfolded proteins in the ER may either be degraded by ERAD or activate the UPR, which induces a set of transcriptional and translational events to the nucleus through activation of 2 transcription factors (ATF6 and XbP1s), an unspliced X-box binding protein1 (XbP1u) and mitochondria via IRE1α/PERK/ATF6α, 3 ER transmembrane protein sensors to restore ER homeostasis via adaptive mechanisms. Conversely, if ER stress persists chronically at high levels, a terminal UPR signal induces cell death via the activation of CHOP/GADD34/DR5/BCL2 when damage is irreversible. Chronic ER stress and defects in UPR signaling are contributors to ND. In addition to UPR, the excessive transfer of Ca^++^ to the mitochondria also leads to enhanced ROS production (oxidative stress) and mitoproteases (BAX/BAK-dependent apoptosome) activation. Depending on the proteostasis context in ND subtypes and their pathological conditions, ERS may trigger distinct signaling pathways. Proteasomes are multicatalytic protease complexes that selectively degrade target proteins into peptide fragments to maintain protein homeostasis.

The majority of the 8–10,000 different proteins expressed in a human neuron require efficient folding in a relevant time scale to develop the three-dimensional tertiary structures that allow correct neuronal functions. The Endoplasmic Reticulum (ER), with its molecular chaperones Heat Shock Protein 70KD (Hsp70s/90s, chaperonins), tetratricopeptide repeat proteins, and nucleotide exchange factors, plays a critical physiological role in the correct folding, post-translational modification, and transport of nascent proteins to their ultimate destinations. Importantly, it requires spastin [108] for its biogenesis. Spastin is a binding partner of Collapsin Response Mediator Protein3/Dihydropyrimidinase-like4 (CRMP3/DPYSL4) and promotes dendritic outgrowth. Mutated spastin, which produces altered neurite structure, is the main cause of Hereditary spastic paraplegia (HSPa) where patients also develop dementia. Overall, precursor cores composed of oligomers provide a template for the reversible attachment of misfolded proteins. The overwhelming accumulation of misfolded/inefficiently-ubiquitylated proteins may overflow in the ER, and generate ER stress (ERS) [49,109], a conserved feature linked to ND. In attempts to remedy the stress, the ER, via Binding Immunoglobin Protein (BiP), activates unfolded protein response (UPR) [2] by stimulating a set of transcriptional/translational programs involving PRKR-like ER kinase (PERK), Inositol-requiring Enzyme 1α (IRE1α), Estrogen Receptor Alpha (ERA), the ubiquitin-proteasome system, molecular chaperones, and Activating Transcription Factor 6 (ATF6) that subsequently initiates the activity of Endoplasmic Reticulum-Associated Degradation (ERAD) [110]. This structure retro-transports misfolded proteins to the cytosol for degradation before they become a threat to cell survival and induce the expression of death-associated proteins, including calpains/caspase-12/B-cell lymphoma-2 (Bcl-2)/C/EBP homologous Protein (CHOP)/Myeloid Leukemia Cell-1 (MCL1)/BLC2-AssociatedX (BAX)/BLC2-Associated-1 (BAK), and X-box Binding Protein-1 (XBP-1). However, with aging, the dynamic proteostasis network experiences decline, and the post-mitotic nature of neurons makes them unable to dilute toxic proteins through cell division. Therefore, persistent ERS may increase neuronal susceptibility to proteostatic imbalance [111], which could account for the accumulation of protein aggregates found in patients diagnosed with ND: i.e., IRE1, ATF6α, PERK, eIF2α, BiP, and CHOP. Overall, the excessive accumulation of toxic misfolded proteins/insoluble aggregates found in ND patients and in animal models may cause dysproteostasis, Ca^++^ dysregulation, ERS, mitochondrial dysfunction, membrane fragmentation, dendritic abnormalities, and ultimately initiation of autophagy/apoptosis (Figure 1).

### 2.1. Proteostasis Imbalance in AD

The neuropathological hallmarks of AD include neuronal and glial dysfunction [112], and the accumulation of hyperphosphorylated tau in Neurofibrillary Tangle (NFT) and amyloid-β plaques associated with the detrimental effects of the ApolipoProtein E allele (*Apoe4*) [77]. In the early stage of AD, the continuous accumulation of Aβ40 and Aβ42 causes mild alterations of ER structure and function, resulting in activation of the UPR pathway in the hippocampus and fronto-temporal cortices. UPR activation increases phosphorylation of three stress sensors IRE1/PERK/ATF6 and eIF2α in AD neurons to eliminate misfolded proteins via translocation to the cytoplasm for degradation whereas the UPR-pro-apoptotic pathway, a potential cause of ND, is activated in ERS [78]. Chaperones (Hsp72/73), Glucose regulated protein 94 (Grp94), Protein Disulfide Isomerase (PDI), BiP, and calreticulin are up-regulated in cerebrospinal fluid (CSF)/brain of AD patients. Furthermore, it has been shown that (1) extra-synaptic calpain can cleave Striatal-enriched Protein Tyrosine Phosphatase (STEP), leading to toxic Nuclear Factor of activated T cells (NFATs) signaling and phosphorylation of tau; a recent study showed that dephosphorylated NFATs were transferred into the nucleus, and the calcineurin-NFATs signaling pathways affect Tau/Aß40–42 activity, which may contribute to AD pathology [113]; (2) proteostasis is altered in AD [78]; and (3) Presenilin (PS), a γ-secretase component, is expressed primarily in the ER with mutations affecting IRE1 and PERK, which are associated with early-onset AD by rendering neurons more susceptible to apoptosis. Overall, accumulating data indicates ERS plays a key role in AD [79].

### 2.2. Proteostasis Imbalance in PD

PD is characterized by a selective loss of Dopaminergic neurons (DAn) in the Substantia Nigra (SN) with misfolding and aggregation of α-syn within the Lewy plaques, mitochondrial dysfunction, and elevated production of Reactive Oxygen Species (ROS) [82]. It is a mainly sporadic progressive form of ND. Approximately 10% of cases are caused by genetic mutation of a variety of genes [83]—including Parkinsonism-associated deglycase 7 (*Park7*/*DJ1*), Leucine Rich Repeat Kinase 2 (*LRRK2*), Synuclein Alpha (*SNCA*), Parkin RBRE3 Ubiquitin Protein Ligase (*PRKN*), ATPase Cation Transporting 13A2 (*ATP13A2*), PTEN-induced Kinase1 (*PINK1*)—which can increase deposition of misfolded proteins. The remaining 90%, categorized as sporadic PD, is of still unknown origin, although it is well known that injection of 1-methyl-4phenyl-1,2,3,6-tetrahydropyridine (MPTP) or 6-hydroxydopamine (6-OHDA) damages DAn. Numerous studies implicate mutated LRRK2/Parkin/PINK1, as well as α-Syn, in the development of PD by triggering ERS and making mitochondria—the other major player in the Ca^++^ regulation via its ability to rapidly sequester large Ca^++^ transients—more vulnerable to oxidative stress [84]. Administration of an ERS inhibitor reduced the α-synucleinopathy effect and protected it from cell death showing that α-Syn is directly involved in ERS.

### 2.3. Proteostasis Imbalance in HD

HD is an inherited autosomal-dominant ND that originates from the expansion of 36 or more repeated CAG trinucleotide sequences. HD patients experience characteristic motor dysfunction. Initially, patients lose their Neostriatal Spiny GABA neurons (nssGABA) projections due to striatal dystrophy, which later spreads throughout the CNS. In contrast to other NDs, mutant Htt (mHtt) has not been found inside the ER and its disturbance of ER homeostasis remains poorly understood [8]. However, reports using in vitro models demonstrated that over-expressed mHtt interacts with Glycoprotein 78 (gp78), an ER membrane-anchored ubiquitin ligase, leading to ERS through IRE1a/PERK/ATF6 activation, which alters axonal transport through activation of c-Junk N-terminal Kinase (JNK3) and phosphorylated kinesin-1 in CNS neurons. Within the cytosol, mHtt aggregates may enhance toxicity whereas, in the nucleus, they sequester other proteins causing transcriptional dysregulation of CHOP, BiP, Homocysteine inducible ER protein with Ubiquitin-like domain1 (HerP), Apoptosis-regulatory Kinase1 (ASK1), and Ribosome Biogenesis regulator 1 Homolog (Rrs1), as found in post-mortem HD brains [9]. A recent report [10] using Knock-in (KI) mice with a full-length Htt-lacking CAG triplets highlighted amelioration of motor deficits and a reduction of Htt aggregates. 

### 2.4. Proteostasis Imbalance in ALS

ALS is a common progressive motor neuron (Mn) disease with a high fatality rate, dominant motor symptoms, and variable cognitive decline [90]. In 10% of inherited forms, several mutated proteins—Superoxide Dismutase1 (SOD1), TDP-43, FUS, and C9ORF72 [91]—are expressed in abnormally folded conformations which aggregate and cause proteostatic imbalance. Moreover, mutated Vesicle-associated Membrane Protein/Synaptobrevin-associated Protein B (VAPB), implicated in late-onset ALS, also causes the accumulation of misfolded proteins in the ER. It has been reported that mSOD1 undergoes conformational changes leading to aggregation and ERS [92]. Additionally, (1) transcriptional analysis of muted Superoxide Dismutase1 (mSOD1) in iPSCs derived from ALS patients showed upregulation of UPR markers [93] and, (2) GGGGCC hexanucleotide expansion of C9ORF72 and ubiquitinated mutations within TDP-43 were identified in patients displaying both Fronto-Temporal Dementia (FTD) and ALS phenotypes, which suggests a genetic overlap between these disorders [94]. Furthermore, it has been reported that UPR sensors ATF6/PERK/IRE1 are increased in the spinal cord of patients with sporadic ALS [92], whereas reduced expression of Major Histocompatibility Complex Class 1 (MHCI) in Mn makes these neurons susceptible to astrocyte-induced cell death [114].

### 2.5. Proteostasis Imbalance in CJD

CJD is associated with neuronal/synaptic loss, microvacuolation, decreased brain weight, ventricular enlargement, and the presence of abnormal Scrapie Prion (PrP^Sc^) which differs from the normal Cellular Prion (PrP^c^) in its three-dimensional β-sheet structure and its abnormal protease resistance. In all forms of CJD, PrP^Sc^ produces multiple conformers, resulting in self-aggregation and self-propagation [100]. Recent studies suggest accumulated PrP^Sc^ in the ER leads to ERS-induced apoptosis via up-regulation of Gastrin-releasing peptides (GRP58/GRP78/GRP94), observed in the cerebral cortex of CJD patients. Additionally, ATF6, PERK, and IRE1 were shown to be involved in CJD [101]. It was suggested that PrP^Sc^ accumulation induced neuronal toxicity through Ca^++^ dyshomeostasis and ERS/caspase activation/autophagy [102]. Intriguingly, there is a lack of clinical correlation between symptoms and PrP^Sc^ levels, suggesting the presence of partially folding structure/intermediate pathological factors [103]. Importantly, it has been shown that the accumulation of misfolded proteins altered Ca^++^ homeostasis.

## 3. Pathology of Calcium Signaling in Major Neurodegenerative Diseases 

**Figure 2:**
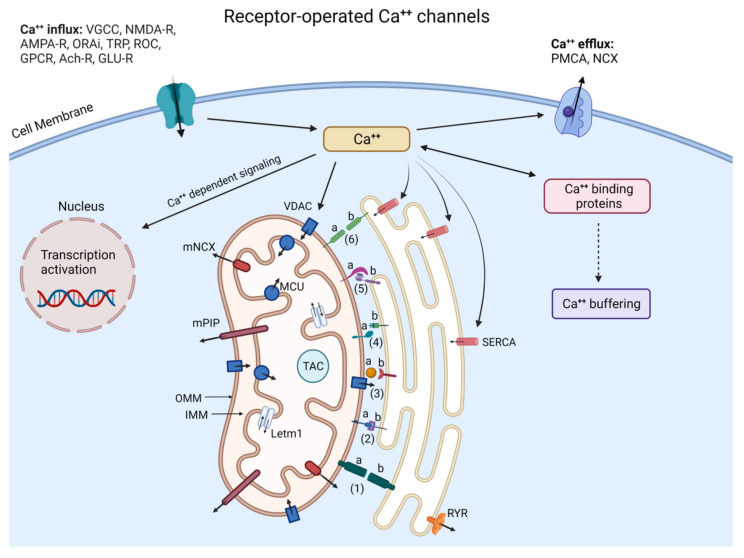
Neuronal Ca^++^ homeostasis via Pore/Receptor-operated Ca^++^ channels, Ca^++^ binding proteins, gene transcription, and ER/Mitochondria interactions. The complex signaling pathways regulating neuronal Ca^++^ concentration and organization require various membrane Ca^++^-conducting channels, intracytoplasmic organelles such as ER and mitochondria, and a great number of Ca^++^ buffering/dependent proteins including calretinin, parvalbumin, calbindin, and kinases. (Ca^++^)_ic_ is determined by the balance between Ca^++^ influx (left side) and efflux (right side) and is buffered in the cytosol by mitochondria through VDAC, MCU, and ER with the help of SERCA while RyR and IP3R mediate ER Ca^++^ efflux. (Ca^++^)_ic_ also regulates the expression of target genes. Ca^++^ released from the ER interacts with mitochondria through MAM and contributes to the activation of the Tricyclic Acid Cycle (TAC) to stimulate ATP synthesis, whereas the excessive transfer of Ca^++^ to mitochondria leads to ROS and BAX/BAK-dependent apoptosome. Inter-organelles do not act as autonomous units but as interconnected hubs that engage in extensive communication through membrane contacts. The proteins within MAM—a central hub involved in different fundamental cell processes—play important roles in maintaining MAM stability, Ca^++^ transport, and apoptosis: 1a, b: REEP1: Receptor expression-enhancing protein 1 (REEP1); 2a: PTPIP51; 2b: VABP; 3a: GrP75; 3b: IP3R; 4a: Protein tyrosine phosphatase interacting protein1 (PTPIP51); 4b: Motile sperm domain-containing protein 2 (MOSPD2); 5a: MFN1; 5b: MFN2; 6a,b: MFN2. Yet, the depletion of the Ʃ−1 receptor leads to abnormal Ca^++^ signaling between ER and mitochondria, and the disruption of ATP production.

Ca^++^ has a critical and diversified role in maintaining neuronal survival. It influences dendritic development, plasticity, synaptogenesis, neurotransmitter release, and is crucial for memory formation [115]. Neurons have extensive machinery to regulate their Ca^++^ homeostasis from influx at the membrane to intracellular release (Figure 2). The ER is the largest store and regulator of intra-neuronal Ca^++^ with its high density of Ca^++^ channels/concentrations regulated by Stromal-interacting Molecules (STIM) and Calcium Release-activated Calcium (CRAC), both of which operate via the Store-operated Calcium Entry (SOCE) pathway [116,117].

Complex downstream Ca^++^ signals require the activity of Calcium Calmodulin dependent protein Kinase (CaMKI-IV), Protein Kinases A/C (PKA/C), Phosphatidylinositol-3-kinase (IP3), Calcineurin (CaN), and calpains which are auto-regulated by Ca^++^. In contrast to very high extracellular Ca^++^concentrations (~1.2mM)**,** cytosolic Ca^++^ levels are maintained in the range of ~200 nM under resting conditions and between 1 and 500 µM upon activation, by calcium-binding proteins and by Sarco/ER Calcium-ATPase (SERCA) [118]. This high differential concentration means calcium can enter neurons through specific Voltage-gated Calcium Channels (VGCCs), and plasma membrane-bound N-Methyl D-Aspartate (NMDA-), Alpha-amino-3-hydroxy-5-methyl-4-isoxazole (AMPA-), Acetylcholine (Ach-), Transient Potential (TRP-) receptors. As no one assay type can guarantee to be the most appropriate, recent in vitro findings [119] and studies in rodents suggest the immediate adverse effects of ERS arise from dysfunction and/or dyshomeostasis of Ca^++^ released into the cytoplasm following activation of Inositol 1,4,5-triphosphate Receptors (InsP3Rs) and Ryanodine Receptors (RyRs). Calcium overload subsequently activates calpains, and, in the case of neurodegeneration, forces neurons to undergo apoptosis via Bcl-2/p53 and caspases. Subsequently, abnormal activation of neuronal calpains may give rise to ND [120]. There is also evidence that: (1) mitochondria are an ATP reservoir and are able to regulate cellular Ca^++^ concentrations via Mitochondrial Calcium Uniporter (MCU) complexes [121,122], glutamate receptors, and L-type Ca^++^ channels; (2) the contact sites between ER and mitochondria at the Mitochondria-associated ER membranes (MAMs) [123] provide direct reciprocal transport between the two organelles through Glucose-regulated Protein 75 (GrP75), Ʃ−1 receptor, B-Cell Receptor-associated protein 31 (BAP31), Mitofusin2 (MFN2), Dynamine-like Protein1 (DNM1L), and other proteins that have emerged as a complex hub fundamental for Ca^++^ homeostasis; and (3) mitochondrial dysfunction due to Ca^++^ overload with ROS triggering superoxide-mediated programmed cell death is observed in ND. These above observations suggest that Ca^++^ dyshomeostasis compromises neuronal well-being and is one of several early causal factors for ND [124]. Indeed, it has been proposed that dysregulated neuronal Ca^++^ is triggered by mutated proteins and that Ca^++^ has an upstream role underlying the pathogenesis of ND [125] (Figure 2).

### 3.1. Dysfunction of Calcium Signaling in AD

In affected brain areas of familial AD patients, Ca^++^ concentrations are increased, primarily due to mitochondrial dysfunction, ERS, and changes in gene expression [126]. Elevated intracellular Calcium (Ca^++^)**_ic_** affects tau phosphorylation and APP processing resulting in the generation of Aβ42, which can further elevate (Ca^++^)**_ic_** through VGCCs and NMDA receptor activation [127]. Other reports mention: (1) the interference of Ca^++^ leakiness from the ER with InsP3Rs/RyRs channels activity [128]; (2) the formation of novel Ca^++^ permeable pores by Presenilin (PS1/PS2); (3) the involvement of calsenilin and calmyrin; (4) the upregulation of ER-mitochondrial contact points; (5) high (Ca^++^)**_ic_** levels leading to unbalanced Calcineurin (CaN) and CaMKII expression. Altered expression of these two synaptic proteins may lead to synaptic loss and memory impairment [129]. Importantly, Ca^++^ dysregulation prior to the emergence of plaques or tangles was found to precede other AD pathologies, such as impaired Amyloid Precursor Protein (APP) processing, tau hyperphosphorylation, and generation of ROS. Collectively, various Ca^++^ signaling mechanisms are dysregulated in AD.

### 3.2. Dysfunction of Calcium Signaling in PD

The involvement of Ca^++^ as a causal factor in PD arises from the following observations: (1) DAn in the SN sustain spontaneous and continuous Ca^++^ influx via L-type CaV 2.3 Ca^++^ channels, making these Dan selectively vulnerable to mitochondrial oxidative stress and undergo a transition of mitochondrial permeability [130]; (2) aberrant expression and/or aggregation of α-syn may enhance Ca^++^ influx from the extracellular compartment [131]; (3) skin fibroblasts of familial PD patients with a mutation of Phospholipase A2 Group 6 (PLA2g6), a protein coded by *PARk14*, displayed depleted ER Ca^++^ stores [132]; (4) the expression of the Ca^++^ buffering protein Calbindin-D28K (CB-D28K) is correlated with Dan vulnerability in PD [133]; and (5) higher concentrations of Ca^++^, Dopamine (DA), and neuromelanin in the SN may contribute to higher susceptibility to l-DOPA-induced neurotoxicity [134]. Finally, medium spiny neurons of the calbindin-poor striosome are more vulnerable than those in the calbindin-rich matrix [135]. Additionally, MAMs that function as ion transfer regions, and involve several proteins—DJ1, α-syn, PINK1, Parkin, Voltage-dependent anion channels (VDAC), Mfn2, Beclin1—are altered in PD. 

### 3.3. Dysfunction of Calcium Signaling in HD

The SOCE pathway is important for the activity of nssGABA striatal neurons and spino-genesis, and is elevated in HD, suggesting a cascade of pathological processes related to alterations in Ca^++^ signaling [136]. This is mainly due to aggregation of toxic mHtt isoforms which: (1) alters various transcription factors and proteins functions including the Ca^++^ signaling components (Calmodulin, calretinin, calmyrin1) [137]; (2) increases expression of NR2B-bearing NMDA-R leading to increased Ca^++^ influx whereas extra-synaptic insertion of NMDA-R activates the pro-death signaling pathway [138]; (3) activates caspase-12 and UPR stress sensors that increase ERS levels [139]; (4) disrupts mitochondrial Ca^++^ homeostasis [140]; and (5) affects the activity of the ER Ca^++^ IP3R and HAP1A [141]. As a result, such dysfunction may be significant for striatal nssGABA neuron activity. Finally, when Ca^++^ stores in the ER are depleted, STIM2 refills ER Ca^++^ by recruiting Orai1 Calcium Released-activated Calcium Modulator 1 (ORAI) channels to allow Ca^++^ entry.

### 3.4. Dysfunction of Calcium Signaling in ALS

Several studies have shown that, in addition to mSOD1, dysproteostasis in the ER [142], disruption of intracellular Ca^++^ homeostasis [143], defects in the Glu-R2 subunit of glutamate AMPA-R [144], and evoked mitochondrial dysfunction are involved in ALS. Furthermore, several different pathogenic mechanisms have been identified: (1) intra-mitochondrial localization of mutant SOD1 has been correlated with mitochondrial dysfunction; (2) levels of ATP/oxygen consumption/respiratory chain enzymes are decreased in Mn cell lines expressing mutant human SOD1; (3) expression of CB-D28K and parvalbumin is significantly decreased in Mn lost in the early stages of ALS [145] whereas calreticulin is downregulated in neuronal models of ALS. Together, these pieces of evidence also point toward a role for calcium dysregulation in the pathogenesis of ALS.

### 3.5. Dysfunction of Calcium Signaling in CJD

Nanomolar concentrations of purified PrP**^Sc^** induce cytoplasmic ER-Ca^++^ release in cultured cells. This Ca^++^ effect occurs because PrP**^Sc^** increases the sensitivity of these cells to cell death by affecting calcium homeostasis mediated through SERCA pumps, RyRs, and IP3Rs calcium channels [146]. Since many ER chaperones and foldases require calcium to maintain optimal activity, this calcium imbalance results in an increase in the percentage of newly synthesized proteins that are misfolded in the ER lumen. The elevated levels of cytosolic calcium hyper-activates mitochondria-dependent apoptotic pathways and brain phosphatase CaN, critical in controlling important signaling events modulating neuronal fate and functioning [102,147,148].

## 4. Selective Neuronal Vulnerability in Major Neurodegenerative Diseases

### 4.1. Dendritic Morphology Underlying Neuronal Vulnerability

The pathological hallmarks of ND include the accumulation of mutated proteins and altered Ca^++^ signaling accompanied by the selective vulnerability of damaged neurons associated with each disease etiology [149,150] (Table 2). Imaging studies in prion-infected animal models have demonstrated that the abnormal dendritic shafts and dendritic spines occur early during the disease course, well before symptoms appear. Degenerated neurites are also found in patients with CJD [151]. Several murine ALS models have shown that vulnerable neuron populations display dendritic alterations before neuronal death. Similar structural and functional alterations are observed in Mn found in human sporadic and familial forms of ALS [152,153,154]. In PD patients and the subacute MPTP model, early loss of dendritic processes and striatal fibers was detected [155]. Relative to the aged-matched control group, AD patients showed reduced dendritic arbors in the parahippocampal formation. Furthermore, dendritic dystrophy emerged prior to the formation of plaques and tangles [156]. A similar dendritic phenotype was found in 4–6 week old 3xTg-AD mice while cognitive impairment was observed at 5–6 months. Finally, in HD, a decrease in Microtubule-associated Protein 2 (MAP2) levels might be related to the early dendritic arborization abnormalities observed in striatal neurons long before symptom onset [157]. Importantly, although pathogenic proteins are ubiquitously expressed, certain neuronal subgroups are relatively resistant to degeneration. For example, neurons of the hippocampus are primarily affected in AD, in the substantia nigra in PD, the spinal cord in ALS, and the striatum in HD. It remains unclear how in AD, the granule neurons in Dentate Gyrus (DG) are spared while pyramidal neurons in the hippocampus are lost, and why in PD, DAn in SN are differentially affected compared to DAn in the Ventral Lateral Area (VTA) [158,159,160,161]. It is likely that early synaptic and dendritic abnormalities may contribute to the initiation of selective neuronal vulnerability.

Notably, dendrite morphology is essential for proper neural circuitry functioning, and dendritic pathology is seen in the early stages of ND neuron dysfunction (i.e., pyramidal and Betz neurons in AD and ALS, respectively) and death suggesting a causal relationship between abnormal dendrites and ND [162]. Dendrites—accounting for more than 75% of neuronal volume—possess a rich array of Ca^++^ channels [154,155] and are key compartments of neuronal information processing; their altered structure and function contribute to neuronal network dysfunction, and consequently, the cognitive impairment observed in ND. It has been shown that extracellular calcium can also directly influence dendritic morphology by activating extracellular calcium-sensing receptors [156]. Importantly, dendritic arbor defects seem to be the outcome of several common combinatorial alterations in local dendritic components including Ca^++^ concentrations [19,163], the cytoskeleton [53], dendritic mitochondria [164,165,166], and Golgi outposts [167].

### 4.2. Intracellular Signaling Mechanisms Underlying Neuronal Vulnerability

Extensive investigation has revealed that different diseases are associated with distinct signaling patterns originating from the accumulation of toxic proteins involved in dendritic dysgenesis as a result of specific regional and neuronal vulnerabilities [162,168]. Some have suggested that such phenotypic heterogeneity may arise from the propagation and variability of specific toxic proteins expressed through different selective connectomic networks or targets. However, despite advances in our understanding of ND, very little is known regarding the mechanisms involved in selective neuronal dendritic vulnerability [169]. For example, in some Transmissible spongiform encephalopathies (TSE), accumulation of misfolded PrP in the brain is not associated with the typical neuropathological changes or any clinical signs of disease [170,171]. Additionally, accumulation of Aβ-amyloid plaques in the absence of clinical symptoms has been observed in the brains of cognitively normal-aged individuals [172]. Furthermore, in ALS, selective neuronal damage does not correlate with the distribution of SOD1 throughout the CNS [173]. Importantly, by the age of 85, 60% of people have plaques, but only 10% develop dementia. Taken together, the direct causal relationship between dysproteostasis and neurodegenerative mechanisms is not fully understood. This suggests that a mutant protein does not necessarily condemn a particular neuron to death and that molecular mechanisms involved in ND are multifactorial. Since misfolded proteins disrupt Ca^++^ homeostasis, which is involved in ND, this mechanism must be considered when developing meaningful therapeutic strategies. In prion disease, PrP**^Sc^** initiates a synaptotoxic signaling cascade that activates NMDA and AMPA receptors in hippocampal neurons, increases intracellular Ca^++^ concentrations, stimulates p38 Mitogen-activated Protein Kinase (MAPK), and depolymerizes actin filaments leading to the collapse of the cytoskeleton. Furthermore, the brains of affected AD patients show increased levels of Ca^++^ and activation of Ca^++^-dependent enzymes [174]. This suggests that Ca^++^ contributes to the development of AD by triggering Ca^++^ release from the ER and mitochondria and expression of Ca^++^-dependent genes [175]. Similarly, in ALS, subgroups of the spinal cord and brainstem Mn have low amounts of Ca^++^ buffering protein expression and develop a toxic Ca^++^ shift between ER/mitochondria that make them vulnerable [176]. Ca^++^ dyshomeostasis is also found in postmortem brain samples of PD/HD/CJD patients, and neurons with high expression of Ca^++^ binding proteins were more resistant to cell death (Table 2). These observations highlight that understanding the relevant signaling players is critical to designing effective treatment.

Indeed, there are many common cellular processes underlying misfolded proteins and Ca^++^ dyshomeostasis across ND: if these processes -including altered dendrites/spines structures, and neuronal vulnerability- are central to ND, then a therapeutic intervention that slows them may also slow and/or delay ND. One such approach, through genetic therapy, may prove efficient in the clinical battle against neurodegenerative diseases.

## 5. CRMP3/DPYSL4 as a Potential Neuroprotective Target for ND

Among the many well-known clinical and molecular features of ND, current evidence emphasizes the role of dendritic dystrophy, [162,177,178,179] which is another common ND feature. Importantly, dendrites which usually carry 80–85% of synaptic junctions are plastic structures and their abnormalities appear to be reversible, making them critical early treatment targets. Different guidance cues—neurotrophins [180], semaphorins [181], and adhesion molecules [182]—are crucial extrinsic regulators of dendrite development.

**Figure 3:**
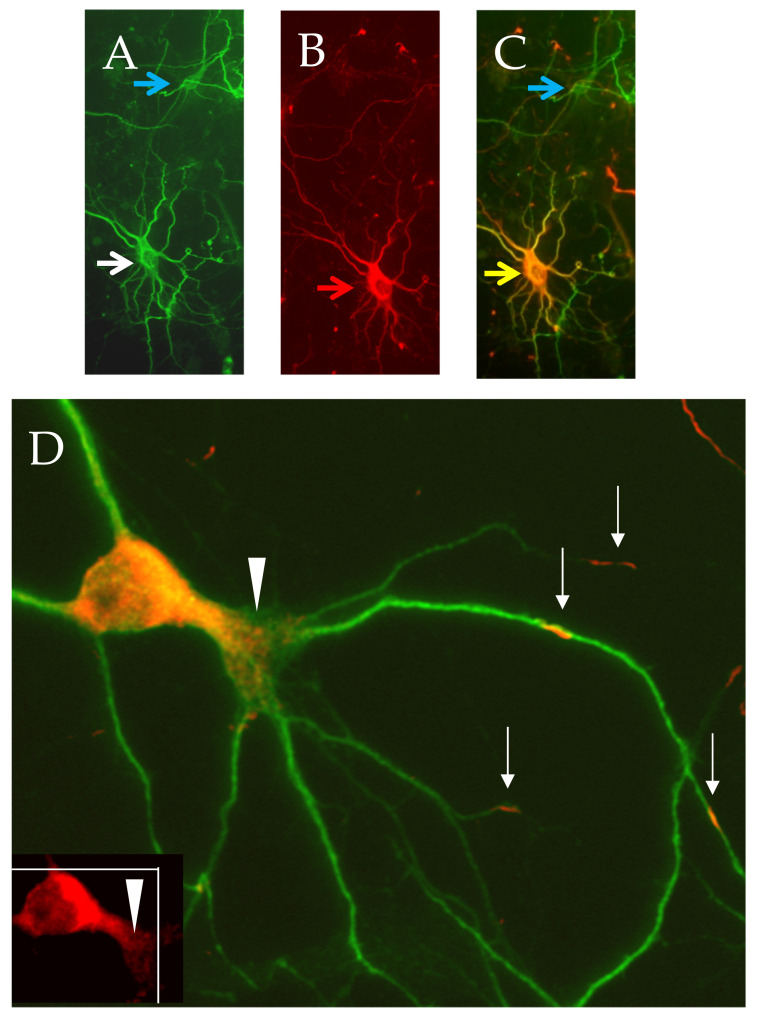
CRMP3-mediated dendritic activity. Representative CRMP3-transfected neurons were immunostained for dendritic marker MAP2 ((**A**) white arrow and Flag-CRMP3; (**B**) red arrow). Overlay images of transfected neurons are in orange ((**C**) yellow arrow; untransfected neuron: blue arrow). Flag-CRMP3 transfected neurons are characterized by an increase in lamellopodial/dendritic formation ((**D**), white arrows heads). Interestingly, the protein did not exhibit passive lateral diffusion but presented as consistent puncta over long distances in the soma and the dendrites with some extending up to the dendritic tips suggesting an active transport of the protein into dendrites ((**D**) white arrows).

In our efforts to explore an effective dendrito-therapeutic strategy, we identified CRMP3/DPYSL4 as a new positive intrinsic signaling regulator of dendrites and their spines in hippocampal neurons. We found that CRMP3/DPYSL4-deficient mice have abnormal dendritic structure, complexity, and correspondingly altered Long Term Potentiation (LTP). Over-expression of CRMP3/DPYSL4 induces lamellipodia formation and dendrite outgrowth (Figure 3) [183,184,185,186].

Our group has also revealed that members of the CRMP family are involved in multiple neurological disorders [187,188,189,190,191,192,193]. Others have shown that CRMP3/DPYSL4 is a phosphoprotein interacting with actin/tubulin in hippocampal neurons [194] and involved in the semaphorin-plexin signaling pathway [195] present in growth cones and the tips of dendrites in mammalian neurons [196]. CRMP3/DPYSL4 is part of a subset of long-lived synaptosome proteins important in memory maintenance [197], displaying dysregulated expression in the brain of AD patients [198], 3xTg-AD mice [199], and in HD iPSC lines. Rosiglitazone, a Peroxisome Proliferator Activated Receptor Gamma (PPARγ) agonist that preserves cognition in insulin-resistant patients with early AD for 4–6 months [200] also improves cognition of Tg2576 AD mice and concomitantly increases hippocampal CRMP3/DPYSL4 expression [201]. Finally, CRMP3/DPYSL4—which is associated with ApoE4 [202]—was suggested as a pre-symptomatic marker and its involvement was investigated during the initiation of AD [203].

Remarkably, in humans, an exuberant dendritic arbor positively correlated with mental capacity [204] while a large-scale Genome-wide Association Study (GWAS) meta-analysis from 269,867 individuals listed *CRMP3/DPYSL4* as a candidate gene correlating with high Intelligence Quotient (IQ) [205], and a linkage disequilibrium pathway analysis showed plexin enrichment in individuals with ~170 IQ [206]. From a clinical perspective, memory impairment is the central problem and most frequent reason for admission into nursing facilities. If the dystrophic dendrite/altered spine phenotype is important in the early physiopathology of ND patients, then cognitive dysfunction may be ameliorated by targeting mechanisms of compensation, remodeling, and/or repair of dendrites. From these observations, and although the mechanisms underlying ND are not completely understood, therapeutic strategies combining multiple treatments to simultaneously rescue dysfunction of ND-associated elements would be beneficial. For example, disease-specific approaches for normalizing dysproteotasis networks and Ca^++^ dyshomeostasis could maximize protection. This could be combined with enhancing the expression of proteins involved in dendritogenesis to rescue neuronal network alterations. Together, these approaches can hold critical value for ND patients—with cognitive impairment [207]—in the early stages of the disease with beneficial consequences on learning and memory.

## 6. Adeno-Associated Virus (AAV)-Mediated Gene Therapy: From Pre-Clinical Studies to Clinical Trials

**Figure 4:**
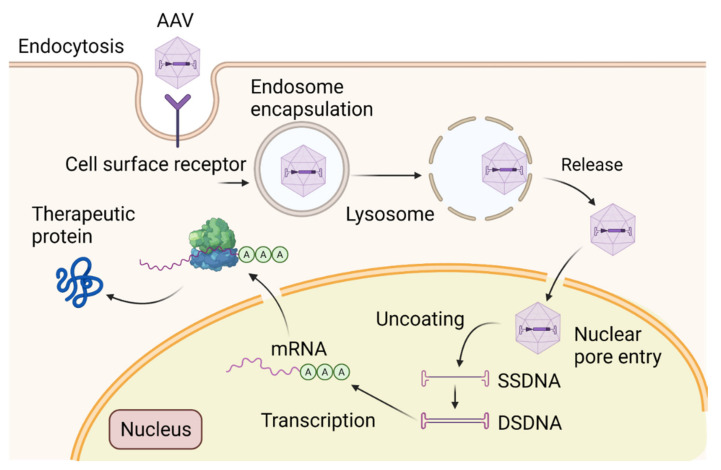
Schematic diagram of the structure of AAV vector mediating gene transfer and therapeutic protein delivery in neurons. Devoid of *rep/cap* genes, the 145 multipalindromic nucleotides of ITR are the only viral origin sequences needed to guide genome replication and packaging during vector production because ITR can form a T-shaped hairpin structure through Watson–Crick base pairing to initiate DNA replication and produce rec-AAVs. Viral ORFs are replaced by a transgene with its regulatory elements. Once the transgene expression cassette is optimized, the next step involves the production of vector stocks. Rec-AAV vectors can be produced at high yields by transient triple transfection in mammalian cells (i.e., HEK293 cells) with three plasmids: the first plasmid containing the transgene of interest, the second plasmid containing *Rep* and *Cap*, and a third plasmid encoding for adenoviral helper genes. The purification of rec-AAV vectors is performed by either column chromatography or gradient centrifugation. Overall, rec-AAV vectors are capable of delivering transgenes into the CNS. They are bound to cell surface receptors, then integrated into the cytosol. Following the endosomal escape, they are uncoated and transported into the nucleus. These single-stranded forms are then converted to double-stranded DNA via host cell DNA polymerases for transcription. This conversion can also be achieved by strand annealing of the plus and minus strands that may coexist in the nucleus. The double-stranded DNA vector can form circularized episomes and persist in the nucleus or undergo integration into the host chromosomes.

Because of the blood-brain barrier and the capacity for achieving persistent gene expression after a single intervention, viral vectors are more efficient at gene transfer in vivo in mammalian CNS cells than liposome carriers which require repeated administrations [208]. These observations have attracted significant interest in the generation of virus-based vectors for preclinical and clinical intervention. The best viral vectors for ND should have high and long-lasting transgene expression, no off-target effects, and elicit no host immune response [209]. Recent successes with gene therapy have indicated the promise of AAV vectors for transgene delivery (Figure 4).

AAV vectors are basically nanoparticles engineered to deliver genetic cargo to the nucleus of a cell. Packed in an icosahedral capsid and with required adenoviral helper genes for the generation of infectious AAV particles, AAV contains a 4.7 Kb single-stranded DNA (ssDNA) coding for *rep* and *cap* genes [210]. These genes are flanked by two inverted terminal repeats (ITRs) necessary for packaging. Being less immunoreactive than other viral vectors (i.e., lentiviruses, retroviruses, adenoviruses, and herpes simplex viruses), and not associated with any known human diseases, they are approved for numerous basic research (i.e., identification of neuronal circuitries functions critical for clinical utility) and clinical trials for ND [211]. Early studies demonstrated different biological properties of different AAV serotypes. Among the multiple AAV1–12 serotypes, AAV9 and AAV2 are remarkable variants: they can cross the blood-brain barrier following intravascular injection and are able to transduce dividing/nondividing cells, respectively [212]. AAV-binding to the cell surface receptors is the first step in the virus infection process, then it undergoes receptor-mediated endocytosis and endosomal trafficking. AAV2 binds to heparin sulfate proteoglycan (HSP), αVß5/α5ß1 integrin, laminin receptor (LR), and IGFR1 [213]. The stability of transgene expression over time depends on specific cell types, AAV serotype, and injection route. These characteristics are considered in cross-packing chimera capsid proteins to optimize cell binding specificity, face hostile environments (AAV2/5) or bear Dynein Motor Complex (AAV-DMC) to increase transgene expression [214]. AAV2 has then emerged as a favorite vector for CNS translational purposes: the replication-deficient virus rarely integrates into the host chromosome and remains episomal, thus reducing the possibility for insertional mutagenesis. For comparison with other serotypes, AAV2 has a small spreading and a strong neuronal tropism. Its genetic cargoes can be DNA/cDNA, silencing/guide/long non-coding/microRNAs, or antisense oligonucleotides. Additional elements—enhancer/intronic sequences/polyadenylation signals—are required for optimal transcription whereas specific promoters are needed for extensive expression in specific cells [215]. Several studies expanded its use to correct a disease phenotype in animal models by (1) gene over-expression to compensate for dysregulated proteins, (2) gene silencing through the use of RNAi/CRISPR-based editing, and (3) gene repair via a knock-in procedure. Gene transfer using AAV vectors has shown therapeutic benefits in several animal preclinical models of RNAi neurological disorders [216,217]. Precisely, (1) Stereotaxic injection of AAV-TRE-CD74 into the hippocampi of TgCRND8-AD mouse, reduced Aβ accumulation in the hippocampus and improved learning and memory [218]; (2) the AAV-Apaf-1-DN-EGFP vector delivery into the striatum of MPTP-treated C57 PD mice prevented nigrostriatal degeneration [219]; (3) striatal injection of AAV5-miHTT-45 prevented mutant HTT aggregate formation in a rat model of HD [220]; (4) CRISPR/Cas9 fused to AAV was able to generate AAV-SaCas9-sgRNAs, and the intracerebroventricular injection of the vector improved the life span of mutant SOD1 transgenic ALS mice [221]. In addition, intravenous injection of AAV9-SOD1-shRNA in SOD1^G93A^, *lox*SOD^G37R^ (carrying human mutant transgene flanked by lox p sites) in non-human primates resulted in a significant reduction of SOD1 protein levels associated with extended survival and slowed disease progression [222].

Regarding clinical trials targeting the CNS, however, several challenges were encountered with AAV gene therapy, especially the need for tightly controlled long-term stable transgene expression. If overexpressed gene products are toxic, gene dosage must be precisely maintained; in addition, the immune response may destroy cells receiving the therapeutic AAV, and preclinical animal models cannot accurately predict the results of gene transfer in humans. Overcoming the impact of the immunological response is complex although a particular route of administration [223] (intra-parenchymal/cerebroventricular/cisternal delivery), re-engineering vector designed to evade the host antibody immune response [224], and an immune-suppression strategy all improve outcome [225]. Despite these technical constraints, advances in understanding the progressive specific/shared impairment in neuronal functions have offered new insights and allowed the development of several clinical trials based on AAV. Human neural tissue fails to regenerate or restore function; therefore, strategies to prevent the loss of function or neuronal cells are critical to ND management. Clinical therapeutic/neuroprotective trials aim to prolong function and prevent the degeneration of neurons. In order to generate meaningful results, clinical trials should ideally optimize the neuroprotective effect in a short-term timeline with appropriate endpoints. Several completed and enrolling clinical trials for ND are based on AAV technology as it is considered a relatively safe and efficient therapeutic tool (Table 3).

The first human trials were implemented more than ten years ago. However, only a few patients have been enrolled yet, and currently, active trials are still Phase I or Phase I/II (safety/proof of concept) trials. There are three main rationales for these gene-based therapies. (1) One intends to promote neuronal survival by promoting local synthesis of factors known to have neurotrophic activity for the neurons specifically altered by the disease with targeted delivery in the area of the brain vulnerable to that disease. For instance, GDNF and neurturin, both from the glial cell-line derived family of neurotrophic factors and shown to markedly enhance dopaminergic neuronal survival, are injected in the putamen and/or substantia nigra for Parkinson’s disease. BDNF, known to be neurotrophic for hippocampal neurons, is tested for Alzheimer’s disease where hippocampal neuronal atrophy is associated with memory deficit. (2) Another proposed mechanism is to increase the level of a missing neurotransmitter by modifying its synthesis. For instance, injecting the DNA of the aromatic amino acid decarboxylase (AADC, Dopa decarboxylase) in the striatum of patients with Parkinson’s disease to increase the conversion of L-Dopa into Dopamine. (3) Another approach is to directly interfere with the pathogenesis of the disease. For instance, the intrathecal injection of AAVrh.10hAPOE2, which expresses human cDNA APOE2 known to be protective against Alzheimer, in patients who are APOE4 homozygotes will establish whether APOE2 will be converted to APOE4 in the CSF. In Huntington’s disease, infusion of engineered AAV5-microRNA (miHTT) into the patient’s striatum so that it binds to HTT mRNA preventing its translation into toxic HTT protein. Taken together, the repertoire of AAV serotypes as vectors is just starting to be explored but may have significant potential in ND treatments.

## 7. Closing Thoughts: Accomplishments and Expectations

Presently, ND gene therapies remain unidimensional, elusive, and limited by the tendency to address one of the pathological changes while ignoring others [226]. Increasing factors that maintain or promote dendritic growth may make cells more resilient to pathological insults and delay the emergence of functional deficits. Considering the intricate signaling cascades underlying ND, the correspondingly complex genetic architecture, and since clinical symptoms are often preceded by a prolonged incubation, an innovative approach could combine combinatorial gene transfer. This approach would include targeting genes to disrupt β-sheet formation, change alternative splicing patterns, fine-tune (Ca^++^)_ic_, and improve dendritic morphology by over-expressing multiple effectors. This could be achieved via AAV-co-transduction or hybrid/dual/triple AAV-vector administration [227], and/or by silencing transcripts associated with pathological stress-activated elements via RNA interference (RNAi) or AAV-CRISPR-Cas systems [228] before the neuronal branching becomes dystrophic and vulnerable cells are lost. Such genetic modifiers which are expected to delay or prevent the onset of ND clinical signs may become a tantalizing potential strategy in the future therapeutic landscape for neurodegenerative disease.

## Figures and Tables

**Table 1:** Overview of clinical symptoms and proteinopathies in major ND. The clinical presentation of ND patients is heterogeneous, although it often has overlapping features such as cognitive impairment. The main specific (limited to a disease) and common (found in several diseases) well-known representative proteins involved in Alzheimer’s, Parkinson’s, Huntington’s diseases, amyotrophic lateral sclerosis, and Creutzfeld-Jakob neurodegenerative disorders are presented. • cjd excluded.

ND	Clinical Signs and Symptoms	Key Protein Candidates	Other Involved Proteins	
			Specificity	Commonality •
HD	Chorea; dystonia; impaired posture, balance and speech; cognitive disorders; learning difficulty.	HUNTINGTIN [8,9,10]	HD71Q [11]; ATN1 [12]; HIP-1 [13]; DMPK [14]; FOXP2 [15]; PRNP [16]	MAP1 [17,18]; Calbindin [19,20]; Calcineurin [21,22]; MAP2 [23,24]; SUBSTANCE P [25,26]; IP3 [27,28]; CDK5 [29,30]; BIP [31,32]; TREM2 [33,34]; IRE1 [35,36]; TNFR1 [37,38]; FGF [39,40]; RAC [41,42]; CDC42 [43,44]; RHO [45,46]; PERK [47,48]; ATF6 [49,50];TDP-43 [51,52]; ACTIN [53,54]; ATXN2 [55,56]; SNCA [57,58]; DCTN1 [59,60]; CTSD [61,62]; GSK3 [63,64]; BACE1 [65,66]; UBQLN2 [67,68]; JNK [69,70]; IFN [71,72]; HSP20 [73,74]; CHOP [75,76].
AD	Memory loss; repeat statements; depression; apathy; irritability; delusions; aggressiveness.	[APP, TAU] [77,78]	[PSEN1, PSEN2, CR1, APOE, PICALM, BIN1, TREM2, ABCA7, PLD3] [79]; [ADAM10, MEF2C] [80]; [UNC5C, AKAP9] [81]	
PD	Motor deficit; often appear asymmetrical; resting tremor; rigidity; dystonia; cognitive disorders.	ALPHA- SYNUCLEIN [82,83,84]	[TAU, LRRK2, SNCA] [85]; [DNAJC6, VPS35] [86]; [VPS13C, PARK2, COMT] [87]; RAB39B [88]; MIR4697 [89]	
ALS	Difficulty walking; weakness in legs and hands; slurred speech; inappropriate laughing; cognitive dysfunction.	SUPEROXIDE DISMUTASE [90,91,92,93,94]	[C9orf72, FUS, VCP, ALS2, SQSTM1, TARDBF] [95]; [KIFA, SETX, OPTN] [96]; [VEGF, ANG] [97]; [CYCLINE F] [98]; [GRN, PRPH] [99]	
CJD	Memory loss; impaired thinking; insomnia; personality changes; jerky movements.	PRION [100,101,102,103]	[COX6, FZD9, RXRG, SOX11] [104]	

**Table 2:** Neuron vulnerability and resistance in major ND. Neurodegenerative disorders are characterized by proteostasis impairment and disruption of Ca^++^ homeostasis and signaling. Despite intrinsically different etiologies, dysregulated Ca^++^ emerged as a common underlying molecular mechanism of altered dendritic structure and neuronal loss in Alzheimer’s, Parkinson’s, Huntington’s diseases, amyotrophic lateral sclerosis, and Creutzfeld-Jakob neurodegenerative disorders suggesting that variations in Ca^++^ buffering protein distribution and (Ca^++^)**_ic_** dyshomeostasis underlies, at least partially, the differential vulnerability.

ND	Neuron Vulnerability		Neuron Resistance	
	Brain Areas *Neuron Types*	Molecular Mechanisms Prevalence	Brain Areas *Neuron Types*	Molecular Mechanisms Prevalence
**AD**	Frontal lobe; Entorhinal cortex; Hippocampus ; Subiculum; Amygdala; Locus Coeruleus; Cingulate gyrus; Nucleus basalis magnocellularis. *Pn. Cn. Ln. RIn.*	Sensitive to oxygen, glucose, energy deprivation, and excitotoxicity. Increased Ca^++^ entry. Low CBP. MisfP	Dentate Gyrus. Cerebellum. *Gn. PurKn expressing* *PcP4. Sn. CI.*	High expression of Y1, calbindin, High CBP as compared to Pn.
**PD**	Striatum; Substantia nigra; Raphe nucleus; Locus nucleus; Limbic cortex; Inferior olivary nucleus; Medulla oblongata; Subiculum. *DAn. Ssn. basal forebrain Cn*.	Neuromelanin, Dp itself. α-Syn. Sensitive to TFs. Mitochondrial-ER dysfunction. Ca^++^ oscillation. Low CBP. Expression of GIRK2. Important CaV1 transcripts.	Ventral Tegmental Area. Pedunculopontin nucleus. *DAn. GABAn. GluTn.*	High expression of calbindin and other CBP. High mitochondrial mass. Undetectable GIRK2 expression.
**ALS**	Spinal cord; Motor neurons network; Brain stem; Corpus callosum; Dentate Gyrus; Entorhinal Cortex; Extrapyramidal alteration. *Cn. ffMn with large axon expressing MMP9. Mn.*	Ca^++^ dysh. Low CBP. Atypical AMPAR. Mutant SOD1 depletes Hsp70/75. EAAT2 high expression. Mitochondrial abnormalities.	Spinal cord *SrMn is more resistant (as compared to ffMn); On and neurons in Onuf’s nuclei.*	*SrMN* expresses Os which binds to αxβ2 integrin (CD11c), its receptor. EAAT2 expression reduced. High expression of calbindin, CBP, and parvalbumin
**HD**	Cerebral cortex; Thalamus; Striatum; Hypothalamus; Cerebellum; Amygdala; Globus pallidus; Putamen; Hippocampus. *EnKn. ParVin. Ssn. SPn. MSn.*	Decreased GDNF, CNTF, BDNF, and CBP. Sensitive to AMPA, NMDA. Increased (Ca^++^)_ic_. Reduction in ATP.	Cerebral cortex. Striatum. *Cn. AsN. SomaTn.*	High expression of parvalbumin, calbindin, and other Ca^++^buffering proteins
**CJD**	Cerebellum; Cerebral cortex; Striatum; Putamen; Thalamus; Corpus callosum. *ParVin. GABAn. ILB4n.*	Increased AMPA, Calcineurin activity, Ca^++^ permeability. Interaction with the α2δ-1 subunit of VGCC. Damage in mitochondria.	Hippocampus. Cerebellum. *Pn, Gn in both areas.*	High expression of calbindin and other CBP.

Abbreviations: Asn: Aspiny neurons; Ca^++^dysh: Ca^++^ dyshomeostasis; CaV1: Membrane Ca^++^ channels; CBP: Calcium-binding proteins; Cn: Cholinergic neurons; CI: Cortical interneurons; DAn: Dopaminergic neurons; DA: Dopamine; EAAT2: Glutamate transporter; EnKn: Enkephalin neurons; ffMn: fast fatigable motor neurons; ILB4n: Isolectin B4 positive neurons; Girk2: G-protein-activated inward rectifier potassium channel 2; GPCR: G-protein-coupled Receptors; GluTn: Glutaminergic neurons; Gn: Granular neurons; GABAn: GABAergic neurons; Letm1: Leucine Zipper And EF-Hand Containing Transmembrane *Protein* 1; Ln: Large neurons; MisfP: Misfolded proteins; MMP9: matrix metalloproteinase-9; Mn: Motor neurons; MSn: Medium spiny neurons; NCX: Sodium/calcium exchanger; ND: Not determined; On: oculomotor neurons; Os: osteopontin; ParVin: Parvalbumin interneurons; PcP4: Purkinje cell protein-4, a putative regulator of calmodulin and CaMKII signaling; Pn: Pyramidal neurons; PurKn: Purkinje neurons; RIn: neurons Reelin-immunoreactive; SPn: Neurons express substance P; Sn: Small neurons; SomaTn: Somatostatin neurons; SrMn: Slow resistant motor neurons; Ssn: Striatal spiny neurons; TFs: Trophic factors (GDNF, BDNF); Y1: Neuropeptide Y1 is involved in intracellular Ca^++^ regulation.

**Table 3:** Strategies for AAV-mediated gene therapy of ND. Sample of clinical trials from clinicaltrial.gov for Alzheimer’s, Parkinson’s, and Huntington’s diseases.

Disease	Clinicaltrial.gov ID	Vectors	Transgenes	Injection Sites
Alzheimer	NCT 05040217	AAV2	BDNF cDNA	Stereotaxic injection into the brain
	NCT03634007	AAVrh.10	APOE2 cDNA	Intrathecal Injection
Parkinson	NCT01621581 NCT04167540	AAV2	GDNF cDNA	Stereotaxic injection into the putamen
	NCT00985517	AAV2	Neurturin cDNA	Substantia nigra and putamen
	NCT01973543 NCT00229736 NCT03562494	AAV2	AADC, Dopa Decarboxylase cDNA	Stereotaxic injection into the striatum
Huntington	NCT05243017	AAV5	HTTmiRNA	Stereotaxic injection into the striatum

## Data Availability

Not applicable.

## Abbreviations

AAV: Adeno-Associated Virus; AD: Alzheimer’s disease; ALS: Amyotrophic Lateral Sclerosis; APP: Amyloid Precursor Protein; ATF6: Activating transcription factor 6; BiP: Binding Immunoglobin Protein; Ca^++^: Calcium; (Ca^++^)**_ic_**: Intracellular Calcium; CaM: Calmodulin; CaN: Calcineurin; CBD: CLN6-Batten Disease; CHOP: C/EBP homologous protein; CLN: Ceroid-lipofuscinosis neuronal; CJD: Creutzfeldt-Jakob’s Disease; CRACK: Ca^++^ Release-activated Ca^++^; CRISPR: Clustered regularly interspaced short palindromic repeated; CRMP3/DPYSL4: Collapsin-response-mediator protein3/Dihydropyrimidinase-like4; CSF: Cerebrospinal Fluid; DA: Dopamine; DAn: Dopaminergic Neurons; DG: Dentate Gyrus; DNM1L: Dynamine-like protein1; eIF2α: Eukaryotic Translation Initiation Factor2α; ERAD: Endoplasmic Reticulum-Associated Degradation; ER: Endoplasmic Reticulum; ERS: ER Stress; FTD: Frontotemporal Dementia; FUS: Fused In Sarcoma RNA Binding Protein; GPCR: G-protein coupled receptor; GrP75: Glucose-regulated protein75; HLA: Human leucocyte antigen; HSPa: Hereditary spastic paraplegia; HD: Huntington’s disease; IMM: Inner mitochondrial membrane; InsP3Rs: Inositol 1,4,5-triphosphate Receptors; IP3R: Inositol Triphosphate3 Receptor; iPSCs: Induced Pluripotent Stem Cells; IQ: Intelligence Quotient; IRE1α: Inositol-requiring Enzyme1α; ITR: Inverted terminal repeats; KI mice: Knock-in mice; LTP: Long Term Potentiation; MAM: Mitochondria-associated ER Membranes; MAPK: p38 Mitogen-activated Protein Kinase; MCU: Mitochondrial Calcium Uniporter; MFN2: Mitofusin 2; MHCI: Major histocompatibility complex class 1; mHtt: Mutant Htt; Mn: Motor Neuron; MOSPD2: Motile sperm domain-containing protein 2; MPTP: 1-methyl-4phenyl-1,2,3,6-tetrahydropyridine; mPTP: Mitochondrial permeability transition pore; NFT: Neurofibrillary Tangle; NOS: Nitrite Oxide Synthase; nssGABA: Neostriatal Spiny GABA neuron; 6-OHDA: 6-hydroxydopamine; OMM: Outer mitochondrial membrane; ORAI1: Orai1 calcium released-activated calcium modulator1; PD: Parkinson’s disease; PERK: PRKR-like ER Kinase; PMCA: Plasma membrane calcium ATPase; PPARγ: Peroxisome Proliferator Activated Receptor Gamma; PrP^c^: Cellular Prion; PrP^sc^: Scrapie Prion; PS: Presenilin; PTPIP51: Protein tyrosine phosphatase interacting protein1; REEP1: Receptor expression-enhancing protein 1; ROC: Receptor-operated channels; ROS: Reactive Oxygen Species; RyRs: Ryanodine Receptors; SERCA: Sarco/ER Calcium-ATPase; shRNA: short hairpin RNA; SMA: spinal muscular atrophy; SN: Substantia Nigra; SOCE: Store-operated Calcium Entry; SOD1: Superoxide Dismutase1; mSOD1: Muted Superoxide Dismutase1; STIM: Stromal-interacting Molecules; TDP-43: Transactive Response DNA Binding Protein 43kDa; TiO**_2_**NPs: Titanium Dioxide Nano-particles; TRPC: Transient receptor potential channel; TSE: Transmissible spongiform encephalopathies; UPR: Unfolded Protein Response; VAPB: Vesicle-associated Membrane Protein/Synaptobrevin-associated Protein B; VDAC: Voltage-dependent anion channels. VGCCs: Voltage-gated Calcium Channels.
